# Defining “elite” status in sport: from chaos to clarity

**DOI:** 10.1007/s12662-021-00737-3

**Published:** 2021-08-05

**Authors:** Alexander B. T. McAuley, Joseph Baker, Adam L. Kelly

**Affiliations:** 1 Faculty of Health, Education and Life Sciences, Birmingham City University, Birmingham, West Midlands UK; 2 Department of Life Sciences, Birmingham City University, City South Campus, Westbourne Road, B15 3TN Edgbaston, UK; 3 School of Kinesiology and Health Science, York University, Toronto, Ontario Canada

**Keywords:** Athlete development, Categorization, Expertise, Measurement, Talent

## Abstract

The past two decades have seen a rapid rise in attention towards talent identification, athlete development and skill acquisition. However, there are important limitations to the evidentiary foundations of this field of research. For instance, variability in describing the performance levels of individuals has made it challenging to draw inferences about inter- and intrapopulation differences. More specifically, recent reviews on high performers in sport have noted considerable variation in how terms such as “elite” are used. This may be particularly concerning for researchers in high-performance disciplines, since they regularly struggle with small sample sizes and rely on research synthesis approaches (i.e. meta-analyses and systematic reviews) to inform evidence-based decisions. In this discussion piece, we (a) highlight issues with the application of current terminology, (b) discuss challenges in conceptualizing an improved framework and (c) provide several recommendations for researchers and practitioners working in this area.

## Introduction

The term “elite” has been used in a wide variety of sporting contexts (Baker et al., 2020). This ranges from samples of “elite” athletes within under nine age groups (an approach that was fiercely condemned (Kirkland & O’Sullivan, 2018)) to those competing at senior international levels (Johnston, Wattie, Schorer, & Bake, 2018). Furthermore, some studies define sub-cohorts within the same sample in relation to their performance relative only to each other (e.g. “elite” versus “non-elite”), rather than in the sport as a whole. Moreover, “elite” status has been inferred based solely on their accumulated training or general experience in a sport, as opposed to being based upon current ability or future potential (Swann, Moran, & Piggott, 2015). Additionally, auxiliary terms such as “super-elite” are becoming increasingly common within scientific literature to classify respective cohorts (Jones, Lawrence, & Hardy, 2018). Collectively, these discrepancies not only undermine the external validity of research investigating the characteristics and prerequisites of “elite performance”, they make it difficult to adequately select studies, draw valid conclusions and generalize findings when conducting research synthesis or translating knowledge into practice. Indeed, as many areas of science are moving towards the analysis of *big data* through involvement in international consortiums and other group-based approaches, standardized definitions of key terms are essential to substantiate the ensuing findings (Williams, Day, & Stebbings, 2017).

In addition to these research-related issues, we need to better understand the *power* the word “elite” can have in both athlete development and performance contexts. Most dictionaries describe “elite” as superior relative to an individual’s peer group. However, the use of this term in practical settings often ignores the possibility (or likelihood) that an athlete’s position may change over time as they and their peer group evolve. It appears the inconsistency of the application of the term “elite” in the scientific literature has also now manifested itself in the practical domain. Anecdotal evidence suggests an English Premier League soccer academy has an “elite under five squad”, who are “treated like pros”, and train more frequently (three times a week) than the other two age-matched groups of adjudged lower ability (Austin, 2019). Indeed, research in youth sport contexts suggests treating young groups in this way has enduring consequences, both for athletes involved and those who are excluded (Bergeron et al., 2015).

In another recent example exacerbated by the coronavirus disease 2019 (COVID-19) pandemic (see Kelly, Erickson, Pierce, & Turnnidge, 2020 for an overview), lockdown restrictions resulted in the suspension of sporting activities below “elite level” in the UK. This caused wide-spread confusion across many communities, as guidelines were vague and applied inconsistently. As an example, one element of the UK government’s definition of an “elite athlete” was the prerequisite of being “aged 16 years or over”. However, in youth soccer, Category One and Two male academies were allowed to continue provision from the under nine age group upwards, whereas Category Three and Four academies were only allowed to continue provision with those aged 16 years and over (The Football Association, 2020). Perhaps more importantly, all female academy provision was suspended at every age group, whilst only the top two leagues in the Women’s Football Pyramid were allowed to continue (compared to the top six in male soccer). Meanwhile, in Northern Ireland, this issue was confounded further, as any athlete competing in a “professional league or competition” was considered “elite”.

These inconsistencies in application are troublesome, as they may send inadvertent messages and build upon pre-existing inequalities and biases throughout organizational structures in sport (e.g. Lawrence, 2017). Indeed, there is a danger that (a) identity foreclosure, (b) early specialization, (c) adult-centred approaches (i.e. treating children as mini adults), (d) a disregard for birthdate, maturation, and survivor biases, (e) a negligence for present and future selection/de-selection well-being repercussions, (f) prioritizing short-term success over long-term development and (g) gender-related biases, are becoming too common in sport. In 2015, the International Olympic Committee (IOC) conducted a critical evaluation of the current state of science and practice of youth athlete development due to similar concerns of the appropriateness and quality of contemporary youth sport experiences (Bergeron et al., 2015). The IOC suggested the *culture* of youth sport in general is excessively both adult and media centred, and there was a need to adopt more viable, evidence-informed, flexible and inclusive frameworks of athlete development. Semantics are fundamental in this regard, as language is an essential part of generating shared practices and forming discourse within specific environments. In youth sport cultures for instance, Kirkland and O’Sullivan (2018) suggest the application of the term “elite” has facilitated the development of an artificial mythology.

## Conceptual challenges

The way we use language matters. However, whilst the solution may be to simply conceptualize an improved framework to more accurately define and group sport participants, this task is not as easy as it appears. Below, we present and discuss several challenges facing researchers attempting to define the competitive playing status of a cohort.

### Individual versus team sport

Whereas individual sports usually measure performance based on quantifiable variables (e.g. distance times, height and length), this is often not possible in team sports where performance is underpinned by a myriad of intrinsic and extrinsic factors, with no isolated performance metric solely capable of measuring overall skill level (Baker, Wattie, & Schorer, 2015). As such, the ability of team sport athletes is commonly inferred from their present competitive playing level, from either an international or national perspective. Although this categorization method appears reasonable, it does not account for the respective disparities in performance standards between countries and leagues around the world. For instance, athletes may represent a country that is towards the bottom of the international rankings or play in the top league of a country where the sport is not popular, less developed and has poorly constructed talent development systems. As such, it is important to assess the level of athletes relative to the competition pool both inside and outside their own country. This particular point is applicable to both individual and team sports. As an example, in Brazil, judo is a very popular and well-developed sport, whereas rugby is not. However, in New Zealand, judo is not particularly popular or well-developed, whereas rugby is. In this context, representing Brazil in judo at the national level is most likely to be more challenging than reaching international status in New Zealand. Similarly, representing New Zealand in rugby at the national level is probably more difficult than reaching international status in Brazil.

### Professionalism

Professionalism has also been used in a dichotomous fashion to differentiate the skill levels of athlete cohorts. For example, in a recent genetic association meta-analysis in soccer (McAuley et al., 2020b), the importance of competitive playing level was assessed via categorizing cohorts as professional and non-professional. Professional cohorts were classified as players that studies specifically described as playing at a professional level, whereas non-professional cohorts were classified as players playing at a semi-professional, amateur or youth level. The authors noted that this particular method of categorization was chosen, as opposed to elite versus non-elite, to circumvent the problematic classification issue of “eliteness” and reduce between-study heterogeneity. However, this crude classification presents similar issues, as there are still substantial differences in performance levels between professional leagues in soccer (e.g. English Premier League versus English Football League Two). This is also the case at the professional level in many individual sports (e.g. competing in golf’s European Tour versus Alps Tour). As such, utilizing a professionalism dichotomy still makes it difficult to objectively quantify the ability of samples and consequently associate findings with specific levels of performance (McAuley et al., 2020b).

### Training time

Although training time is generally associated with higher levels of performance (Baker & Young, 2014), completing a defined number of accumulated training hours (e.g. 10,000) or years (e.g. 10) does not automatically confer “elite” status. There is vast variation in the amount of time it takes athletes to reach the pinnacle of their sport. For example, in most sporting domains the development of high performance takes between 5–30 years, and the highest performers have often achieved the greatest performance of their careers between the ages of 15 and 40 years (Ericsson & Harwell, 2019). Consequently, international-level performers have accumulated between 3000 and 40,000 h of engagement in domain-specific activities, before producing their peak performance (Baker & Young, 2014). In addition, we must consider the confounding effects of *age* on the development of performance and acquisition of higher levels of skill. As Baker et al. (2015) suggest, if we view athlete development as a continuous process of adaptation, then there is inevitably going to be a strong relationship between level of performance and athlete age, especially given the relationship between training hours and age (see Baker & Young, 2014 for a review). Therefore, researchers should avoid using an individual’s training time, or even experience, in a specific sport as criteria to infer proficiency, given the clear inter-individual heterogeneity that exists.

### Youth athletes

Labelling youth athletes as “elite” may be problematic for a number of reasons. For instance, athletes who belong to and progress in talent development systems are seen to possess the potential to reach the pinnacle of athletic performance within their domain when they are older. However, there is no assurance they will ever actualize this projected outcome of their development. In contrast, it is not uncommon for a youth athlete previously adjudged to have less potential to reach the pinnacle of athletic performance when they are older. Current talent predicting models and methods are generally inaccurate and unreliable (Baker, Schorer, & Wattie, 2018) because they are based upon indicators of early performance success, which are poor indicators of performance success at adulthood. This issue is amplified further when we consider that time is inversely related to accuracy in prediction models (Till & Baker, 2020). As such, the earlier identification and selection procedures are applied to youth athletes, the more inaccurate projections concerning their development will be. Talent is not a fixed capacity and evolves dynamically over time; therefore many of the qualities that distinguish top athletic performance in adults may only emerge later in development (Baker, Wattie, & Schorer, 2019; McAuley, Baker, & Kelly, 2021). Thus, endeavors to identify these qualities early in an athlete’s development are problematic because they do not account for these changes. Furthermore, as the demands and characteristics that encompass optimal performance within specific sports alter unpredictably over time, what we regard as high performance now may be appreciably different in the future (Baker et al., 2018; Till & Baker, 2020).

## Explaining the dissonance

Given the challenges noted above, it may not be surprising there is no general consensus on what “elite” means, as well as how it should be applied to distinguish athletes of different competitive playing levels (Williams et al., 2017). In addition, there may be philosophical explanations; for instance, the absence of irrefutable definitions for key terms in sporting expertise has been identified in broader psychological domains and has been referred to as *language games*. The term language games was originally conceived by philosopher Ludwig Wittgenstein, who advocated that linguistic confusions (i.e. the misuse and misunderstanding of language) were the root of all philosophical problems (Wittgenstein, 1958). However, Wittgenstein (1958) further noted that language games are an unintentional and inevitable facet of human behaviour. Indeed, in the broad psychological literature that exists today, one of the main fallacies remains the varied use of undefined or vaguely defined words (Lourenço, 2001). According to the philosopher Thomas Kuhn, this is *normal science*, as researchers’ activities can be characterized as *puzzle solving* within *paradigms *that have a shared view on beliefs, language usage and meaning (Kuhn, 1962).

On the contrary, paradigms are fundamentally dogmatic and incommensurable, and as such, researchers of differing paradigms are rarely capable of taking part in sensible debate (Feyerabend, 1975). It has been reported that researchers within youth athlete development tend to work within paradigms. More specifically, Dohme, Backhouse, Piggott, & Morgan (2017) found that psychological terms within youth athlete development are only used consistently if an author is involved in more than one paper, and that authors often dismiss their responsibility to clearly explain topic-specific vocabulary. Collectively, this research may explain, to an extent, why practitioners, the media, and policy makers misconstrue the meanings of terms and use them out of context (e.g. “elite”), as they have their capacity to interpret and implement research findings obscured by differing research paradigms.

These explanations are not excuses for inaction. Indeed, a necessary first step is clarifying how samples are defined and described. Swann et al. (2015) proposed a five variable framework to help researchers evaluate and classify the level of “eliteness” within their samples. Each variable is allocated a score between 1–4, which is summed, and then transformed via a mathematical equation to produce a final score from 1–16. The classification system consists of four categories: (a) semi-elite (1–4), (b) competitive-elite (4–8), (c) successful-elite (8–12) and (d) world-class elite (12–16). Similar classification research has been conducted in physiology. For instance, De Pauw et al. (2013) constructed a classification system for male cycling and introduced the neutral term *performance levels 1 to 5*, representing untrained, recreationally trained, trained, well-trained and professional subject groups, respectively. This approach was later replicated and applied to female cycling research (see Decroix, De Pauw, Foster, & Meeussen, 2016). Whilst the variables, scores and weightings of these approaches can clearly be debated, they provide useful devices to encourage researchers to carefully contemplate how to describe the performance status of their samples (see a similar approach taken by Baker et al., 2015 for classifying skill levels in sport). However, all of these approaches add to the abundance of terms that have been used in various other taxonomies to describe various levels of expertise, as well as failing to provide recommendations on the term “elite” itself. This is possibly the most important aspect, as “elite” is widely used in academic and practical settings to describe higher performing athletes.

## A call for transparency

Given the aforementioned research in this area, there is unlikely to be external consensus on a new universal definition, at least in the near future. Moreover, a generalizable definition across all sports is not feasible due to the complexity of intra- and intersport differences. However, there is a way forward for our field that would accommodate many of the challenges noted above. In our opinion, the best solution is to call for greater *transparency *in describing samples. Not only do we advocate that authors be more explicit in describing the characteristics and rationale for the categorization of their samples, we encourage journal editors and reviewers to demand greater detail in sample descriptions. To be specific, researchers should be required to present specific information concerning all cohort elements that are relevant to athlete status categorization, such as: (a) age, (b) competition level, (c) league status, (d) gender, (e) international ranking, (f) nationality, (g) province/state, (h) sport and (i) success/achievements. These recommendations may seem obvious, but we would note that a recent scoping review of talent research found that ~15% of studies did not report gender and 22% did not report nationality (Baker et al., 2020). Indeed, inadequate reporting of participant characteristics is a common finding throughout research syntheses in sport (McAuley et al., 2020a). Overall, this greater transparency will allow readers to determine their *personal* classifications for samples more effectively, and thus facilitate improved research synthesis and practical interpretations.

In addition to improving methodologies, understanding the influence different words have on key psychological variables (e.g. motivation, perceived competence, locus of control and self-efficacy) is an important area of future research. Current hot button topics such as “mindset” are grounded in the assumption that athletes’ long-term engagement and development are affected by these subtle messages (Wattie & Baker, 2017). However, questions remain concerning the immediate-, short- and long-term implications of using terms like “elite” to describe developing young athletes. These enquiries will be critical to understanding the athlete development process, and remain under-examined by researchers in this area. Indeed, the implications for coaching and coach education would be considerable. Additional research is also required to determine how best to disseminate research findings with key stakeholders (i.e. coaches, practitioners and policy makers) to improve their implementation (Fullagar, McCall, Impelizzieri, Favero, & Coutts, 2019).

## Conclusion

The term “elite” has been inconsistently applied in research, undermining the external validity of findings regarding the characteristics and prerequisites of high performance. Furthermore, the way the word “elite” is applied in practical contexts can be problematic, and may send inadvertent messages and exacerbate existing biases in sport. There is unlikely to be external consensus on a new all-encompassing definition of “elite” in the immediate future. As a result, we call for greater transparency when describing samples to allow readers to determine their personal classifications more effectively, and consequently facilitate improved research synthesis and practical interpretations.

## Declarations

For this article no studies with human participants or animals were performed by any of the authors. All studies performed were in accordance with the ethical standards indicated in each case.
